# Nutrition-dependent phenotypes affect sexual selection in a ladybird

**DOI:** 10.1038/srep13111

**Published:** 2015-08-13

**Authors:** Jiaqin Xie, Patrick De Clercq, Yuhong Zhang, Hongsheng Wu, Chang Pan, Hong Pang

**Affiliations:** 1 State Key Laboratory of Biocontrol, Key Laboratory of Biodiversity Dynamics and Conservation of Guangdong Higher Education Institute, College of Ecology and Evolution, Sun Yat-sen University, Guangzhou 510275, China; 2 Department of Crop Protection, Faculty of Bioscience Engineering, Ghent University, Gent 9000, Belgium; 3 Guangdong Entomological Institute, Guangzhou 510260, China

## Abstract

Environmental factors play a crucial role in influencing sexual selection in insects and the evolution of their mating systems. Although it has been reported that sexual selection in insects may change in response to varying environments, the reason for these changes remains poorly understood. Here, we focus on the mate selection process of a ladybird, *Cryptolaemus montrouzieri*, when experiencing low- and high-nutrition diet regimes both in its larval and adult stages. We found that female ladybirds preferred to mate with males reared under high-nutrition diet regimes, regardless of the nutritional conditions they experienced during their own larval stages, indicating that mate choice of female *C. montrouzieri* is non-random and phenotype-dependent. Such mate choice may depend on visual cues (body or genitalia size) and/or chemical cues (pheromones). Further, females from high-nutrition larval diet regimes produced more eggs than those from low-nutrition larval diet regimes. In addition, diet regimes during adulthood also exerted strong effects on egg production. In summary, our study provides new insight into the mate choice of *C. montrouzieri* as affected by seasonal changes in resources, and suggests that food availability may be a driving force in mate choice.

Sexual selection arises from differences in reproductive success caused by competition over mates^1^,^2^. However, mate choice could be variable due to the candidates’ attractiveness or mate competition, which results in sexual selection^3^,^4^. Hence, fluctuations in mate choice are common in insects, and individuals present the best mating tactics depending on certain conditions^4^,^5^. In order to choose the best potential partner, females can use a number of honest signals, with which the candidates display their qualities or attractiveness^2^,^6^,^7^. Honest cues such as body size, songs or colors are used by insects to attract potential mating partners^1^,^7^,^8^. For example, females of the two-spot ladybird, *Adalia bipunctata*, displayed mating preference for melanic males over non-melanic males^9^; females of the harlequin ladybird, *Harmonia axyridis,* have also been noted to prefer melanic males over succinic ones, which improved the females’ fitness^10^. Further, females of the butterfly, *Bicyclus anynana,* have been noted to choose a mate depending on the characteristics of the wing eyespots^11^. Although previous work has provided insights into the factors affecting female mating preferences^3^,^4^,^6^, it is less well known how nutrition-dependent phenotypes influence female mate choice in insects.

Female insects may gain direct benefits (material benefits), indirect benefits (genetic benefits) or both through mate choice, which improve reproductive performance and/or offspring fitness^2^,^12^,^13^. Direct benefits can be gained via the transfer of a spermatophore or other nuptial gifts, while indirect benefits can be gained by mating with multiple males or with the male having “good genes”^13^,^14^. Such mating benefits may be a factor contributing to female mate choice^1^,^15^. Recent studies have indicated that the spatial dispersion pattern of key resources and sex ratio may also greatly impact on female mate choice^2^,^3^,^4^.

The ladybird, *C. montrouzieri*, is native to Australia and has been introduced in at least 64 countries or territories for the biological control of mealybugs in agricultural crops^16^,^17^. Its mating behaviours have been observed in a previous study indicating that females prefer to mate with males they have mated with before^18^, but there is no specific evidence of what factors underlie such choice. Among other factors, the quality of nutritional resources is a key factor determining mating behavior and female fitness^5^. Insect larvae confronted with poor nutritional conditions often take longer to develop to the adult stage and have a smaller body size at emergence as compared to their counterparts experiencing abundant nutritional resources^19^. Furthermore, recent studies have shown that larval nutritional conditions (in terms of protein and nitrogen levels) can affect adult immune activities and body biochemical composition in insects^20^,^21^. Effects of extreme conditions (e.g. exposure to cold or pesticides) on metabolic rates and immune resistance have also been revealed in adult *C. montrouzieri*^22^.

To demonstrate the relationships between natural resources and female mate selection, in this laboratory study we manipulated the nutritional conditions of *C. montrouzieri* by subjecting the predator to low- and high-nutrition diet regimes both in its larval and adult stages. We first tested the influence of diet on the phenotype of individual ladybirds, and then investigated whether mate selection was influenced by nutrition-dependent phenotypes. We examined how larval diet regimes affect adult phenotypic traits, and investigated how the resulting phenotypes influence female pre-copulatory mate selection. We also assessed reproductive success under varying diet regimes during adult life following mating with different phenotypes of males. We expected that adults would have different phenotypes in terms of body size and immune activities after experiencing a varying diet regime. In order to evaluate adult phenotype and immunity status after experiencing low- and high-nutrition diet regimes during the larval stages, we used body width as a measure of female and male body size, and also measured the size of adult genitalia upon dissection. Further, we investigated immunity of males by measuring the quantitative expression of immune-related genes using qPCR, considering this trait to be an indirect measure of male quality^14^. Subsequently, we examined whether the presence of a “monitoring” male (i.e., a situation where a female and male can see but cannot reach each other) affected mating success of female *C. montrouzieri* when the female was paired with a male. Female mating choice was also studied when simultaneously confronted with two males, which had either experienced a high- or low-nutrition larval diet regime. Finally, we examined the reproductive success of females after mating with different male phenotypes under a varying diet regime.

## Results

### Adult phenotypes

**Figure 1:**
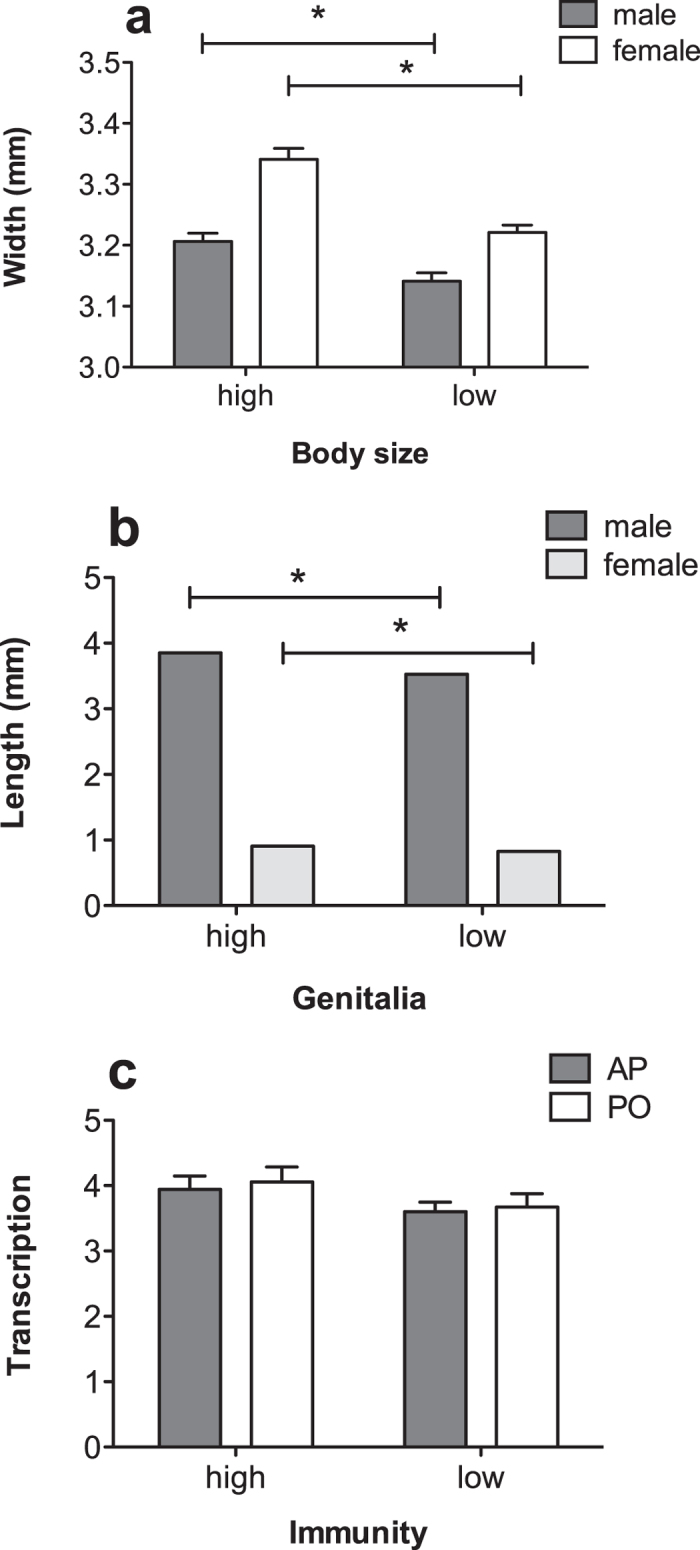
Effects of low- and high-nutrition diet regimes during larval stages of *C. montrouzieri* on phenotypic traits in adulthood. (**A**) body width of male and female adults; (**B**) length of male sipho and female ovipositor; (**C**) quantitative expression of immune-related genes acid phosphatase (AP) and pro-phenol oxidase (PO). Asterisks (*) indicate significant differences. Error bars represent 1 SE value.

Adult body width and size of male and female genitalia were significantly affected by the nutritional conditions during larval stages. Adults from the high-nutrition diet regime had larger body sizes than those from the low-nutrition diet regime (female, F_1, 38_ = 30.714, p < 0.001; male, F_1, 38_ = 11.709, p = 0.002; Fig. 1a). Similarly, adult males and females which had experienced a high-nutrition diet regime had longer genitalia than their counterparts which had developed under a low-nutrition diet regime (female, F_1, 38_ = 33.026, p < 0.001; male, F_1, 38_ = 138.971, p < 0.001; Fig. 1b).

The quantitative expression of acid phosphatase and pro-phenol oxidase in adults from the high-nutrition diet regime was, however, not significantly different from that of adults from the low-nutrition diet regime (acid phosphatase, F_1, 16_ = 1.820, p = 0.196; pro-phenol oxidase, F_1, 16_ = 1.576, p = 0.227; Fig. 1c).

### Mate choice

**Figure 2:**
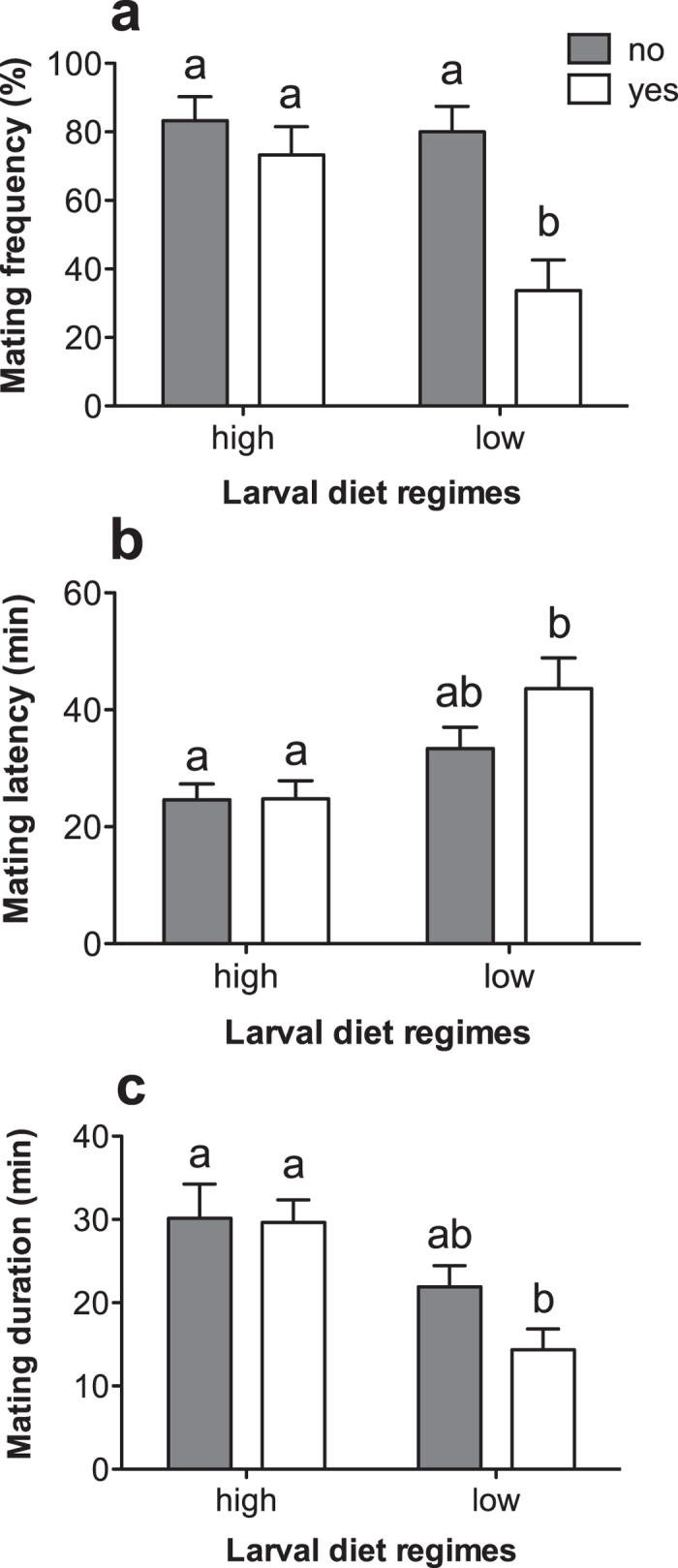
Mating frequency, mating latency and mating duration of females from the high- or low-nutrition larval diet regimes paired with males from their own treatment group, and monitored (yes) or not (no) by a male from the opposite treatment group. Error bars represent 1 SE value.

In the first experiment, when a monitoring male from the high-nutrition larval diet regime was placed in the opposite compartment, mating frequency of females from the low-nutrition diet regime placed together with a male from the same diet regime was lower than for females without a monitoring male (F_3, 117_ = 7.390, p < 0.001, Fig. 2a). In contrast, the mating frequency of females from the high-nutrition larval diet regime paired with a male from the same larval diet regime and presented with a monitoring male from the low-nutrition diet regime, was not different from that of their counterparts having no monitoring male (Tukey test, p = 0.808, Fig. 2a). Further, the copulation latency time of females from the low-nutrition diet regime paired with a male from the same diet regime was affected by the presence of a monitoring male from the high diet regime; this was not the case for females from the high-nutrition diet regime paired with a male from high-nutrition diet regime and presented with a monitoring male from the low diet regime (F_3, 79_ = 4.802, p = 0.004, Fig. 2b). However, the copulation duration of females from the low-nutrition diet regime was shorter when having a monitoring male from the high-nutrition diet regime than that of females from the high-nutrition diet regime with or without a monitoring male (F_3, 79_ = 3.800, p = 0.013, Fig. 2c).

**Figure 3:**
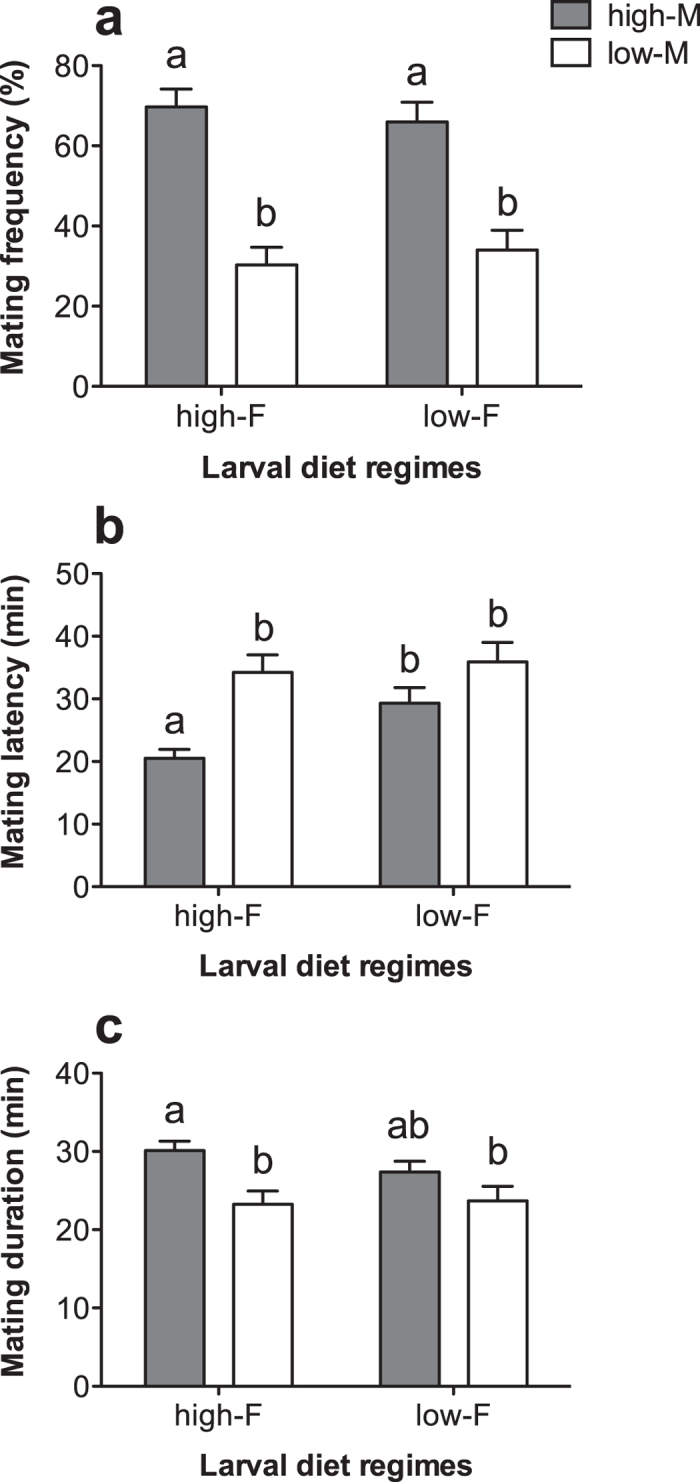
Mate choice of individual females from high- or low-nutrition diet regimes placed together with two males, one from the high-nutrition diet regime (‘high-M’) and the other from the low-nutrition diet regime (‘low-M’), and mating latency and duration after females had made a choice for a particular male. Error bars represent 1 SE value.

In the second mate choice experiment, significantly more females chose to mate with males from the high-nutrition diet regime, regardless of the diet regime they had experienced themselves (F_3, 402_ = 20.152, p < 0.001, Fig. 3a). The copulation latency of females from the high-nutrition diet regime when mating with a male from the same regime was lower as compared with those mating with a male from the low-nutrition diet regime (F_3, 199_ = 9.539, p < 0.001, Fig. 3b). In contrast, copulation duration was longer in females from the high-nutrition diet regime than in those from the low-nutrition diet regime (F_3, 199_ = 4.673, p = 0.004, Fig. 3c).

### Reproductive success

**Figure 4:**
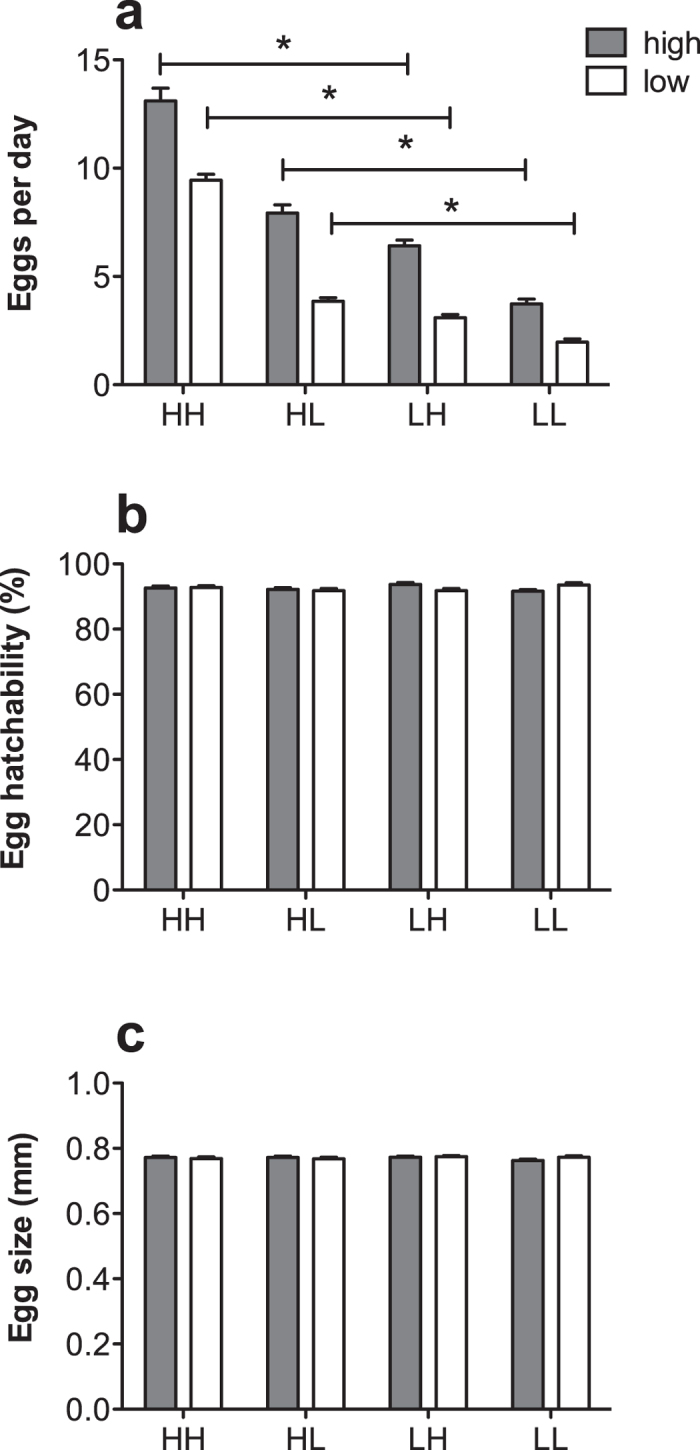
Fecundity, egg hatch and egg size of females experiencing low- (white bars) or high-nutrition (grey bars) adult diet regimes after having mated with a male from high- or low-nutrition larval diet regimes. HH, females from the high-nutrition larval diet regime mated with a male from the high-nutrition larval diet regime; HL, females from the high-nutrition larval diet regime mated with a male from the low-nutrition larval diet regime; LH, females from the low-nutrition larval diet regime mated with a male from the high-nutrition larval diet regime; LL, females from the low-nutrition larval diet regime mated with a male from the low-nutrition larval diet regime. Asterisks (*) indicate significant differences. Error bars represent 1 SE value.

Female adults experiencing the high-nutrition diet regime as larvae had a higher egg production than those which had developed under the low-nutrition larval diet regime, regardless of the larval feeding regime of the males (F_1, 229_ = 27.785, p < 0.001; Fig. 4, table 1). Similarly, females reared under the high-nutrition adult diet regime also had a greater egg production than those reared under the low-nutrition adult diet regime, irrespective of the male larval feeding regime (F_1, 229_ = 16.681, p < 0.001; Fig. 4, table 1). However, the interaction between female size and adult nutrition was not significant (F_1, 229_ = 0.065, p = 0.799; table 1). Further, copulation duration had a significant effect on egg production, as had the three way interaction between male size, mating latency, and copulation duration (F_1, 229_ = 6.75, p = 0.01; table 1). In contrast, female size, adult nutrition and copulation duration had no effects on egg hatchability or egg size (table 2 and 3).

## Discussion

In our laboratory study, the nutritional conditions of the ladybird *C. montrouzieri* experienced in its larval and adult stages strongly affected the mate choice and subsequent reproductive investment. Females preferred to mate with males from the high-nutrition larval diet regime, regardless of the nutritional conditions they had experienced as larvae themselves. Females with larger body sizes (reflecting the larval food regime) had a higher egg production than those with smaller body sizes. Further, adult diet regime significantly affected female egg production. By contrast, both female body size and diet regime during adulthood had no effects on egg hatchability or egg size. Apart from the effects of diet regimes on fecundity, we found that mating duration also had a marginal effect on egg production.

Size parameters of adult *C. montrouzieri* were largely dependent on larval nutrition, indicating that plastic phenotypes of *C. montrouzieri* occurred as a result of varying larval diet conditions. Both male and female ladybirds experiencing food abundance during their larval stages had larger body size parameters than those which developed under conditions of nutrient stress. Effects of environmental conditions experienced during the larval stages on body size and fitness of adult insects have been widely reported in the literature^19^,^23^. For example, larger females of predaceous ladybirds (Coccinellidae) have been noted to have better reproductive performance than their smaller counterparts^24^. On the other hand, size of genitalia plays an important role in mating behavior^25^. Previous studies found that males with larger genitalia are more attractive to females and show a higher mating frequency as compared with males with smaller genitalia^25^,^26^. Besides food availability and individual attractiveness, other environmental factors, like population density, health status or climatic stresses, may also affect the condition of potential mates and resulting mate selection^3^,^4^,^14^.

When examining the expression of acid phosphatase and pro-phenol oxidase in adult *C. montrouzieri*, the expression of these immunity related genes was slightly higher in adults from the high-nutrition diet regime than in those from the low-nutrition regime. Differences were, however, not statistically significant, which may be related to the relatively low sample size. Further research is warranted to verify the effects of food availability on immunocompetence in *C. montrouzieri*. Generally, higher immune activities of well-fed insects are expected to allow them to better resist to environmental stresses, including harmful chemicals such as insecticides or pathogenic microorganisms and parasites^27^,^28^. Previous studies have reported that males with higher immunocompetence may be more attractive to female mates based on different pheromone expression^14^,^29^,^30^. Although our findings indicate that the greater body size of adult males provided them with better mating chances, suggesting that this trait may function as a cue affecting female mate choice in this ladybird, it remains unknown whether other factors, like body coloration, also play a role.

In our first mate choice experiment, the presence of a monitoring male from the high-nutrition larval food regime affected pre-copulatory mate selection by females when paired with a male from the low-nutrition larval food regime. By contrast, female mating choice was not affected by a monitoring male if the female was paired with a male that had developed under the high-nutrition larval food regime. In the first situation, the presence of a monitoring male affected mating frequency, as well as mating latency and duration. This finding indicates that male body size and/or pheromones may be important cues determining the mate choice by female *C. montrouzieri*. A few studies have also reported that mate choice in ladybirds is not random but phenotype-related, and that females exhibit preferences to mate with males possessing specific traits (e.g. *H. axyridis* females preferring to mate with melanic males; *C. montrouzieri* females preferring to mate with males with which they had mated before)^10^,^18^.

In our second mate choice experiment, we found that females preferred to mate with males that had experienced the high-nutrition larval food regime, regardless of their own feeding history. This phenomenon may again be explained both by visual and chemical cues emitted by the males and possibly be related with the higher immunocompetence of larger males as compared with their smaller counterparts. However, the exact mechanisms behind female mate choices in *C. montrouzieri* remain unknown. A recent study revealed that cuticle hydrocarbons are an important determinant in mate choice of crickets, which has provided more insight into the understanding of these mechanisms^31^. Further, competition among males may have also had an influence on the outcome of pre-copulatory events in our experiment. Although a few studies have found that male-male competition in insects is a strong factor affecting female mate choice, with stronger males having more mating chances than their weaker competitors^4^,^32^,^33^, we observed that female *C. montrouzieri* refused mating approaches by aggressive or undesirable males by spreading their wings and flying away.

We also found that adult food regime greatly influenced female egg production but had no effects on hatching rates or the size of the eggs produced. Females experiencing food abundance during adult life produced more eggs than those confronted with low food levels. Adult nutritional conditions may affect reproductive success by mediating survival maintenance and reproduction^34^,^35^. In a resource-barren environment, insects may allocate major resources to survival maintenance rather than to reproductive success^34^,^36^. Although in our study hatching rate or egg size were not affected by adult nutritional conditions, in other insects egg size has been found to be smaller when parents were confronted with unfavorable food conditions^35^,^36^. Furthermore, in our study egg production was also affected by female body size, indicating that a female’s phenotype may affect its reproductive performance. A previous mating experiment with *C. montrouzieri* showed that multiple mating with a male also increases the egg production of females^18^.

In conclusion, by investigating the net effects of varying diet regimes of *C. montrouzieri* on adult phenotypes, nutrition-dependent mate choice and reproductive success, it was revealed that the nutritional conditions of *C. montrouzieri* experienced both in its larval and adult stages may directly or indirectly influence mate choice and reproductive performance of the predator. These findings may provide new insights into why and how mate choice of *C. montrouzieri* may vary with seasonal changes in resources. Further research is warranted to unravel the underlying mechanisms of mate choices in *C. montrouzieri* and how they affect long-term population dynamics.

## Methods

### Adult phenotypes

*Cryptolaemus montrouzieri* used in this study were derived from a laboratory colony maintained at Sun Yat-Sen University, Guangzhou, China, since 2006. The ladybirds had originally been collected from India and were reared according to a semi-natural system at ambient laboratory conditions (T = 25 ± 1 °C, RH = 60 ± 10%). Larvae and adults of the ladybird were fed on citrus mealybugs, *Planococcus citri* Risso (Hemiptera: Pseudococcidae) maintained on pumpkin fruits (*Cucurbita moschata* (Duch. ex Lam.) Duch. ex Poiretand). Prior to the experiments, female and male adults were randomly collected from rearing cages and 50 pairs were paired in Petri dishes to oviposit. Eggs laid on the third day were collected and allowed to hatch. Emerging larvae were used in the experiments.

**Figure 5:**
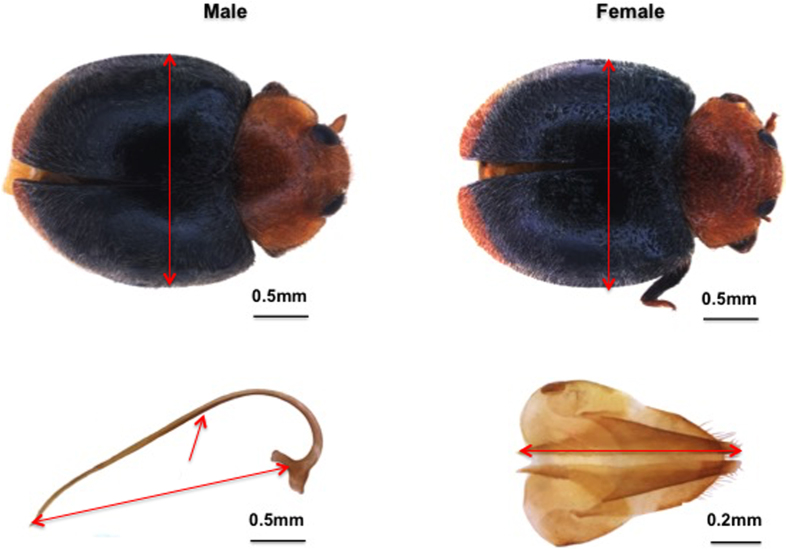
General morphology of male and female adults of *C. montrouzieri*, and details of male sipho (bottom left) and female ovipositor (bottom right). Arrows indicate where measurements of body width and length of sipho and ovipositor were measured (photo author).

To examine how larval nutrition influenced adult phenotypes and immunity, newly emerged larvae were subjected to one of two diet regimes, simulating conditions of food abundance and nutritional stress. In the high-nutrition diet regime, 10 larval *P. citri* (ca. 1.5 mm long) were supplied to each Petri dish and refreshed daily; in the low-nutrition diet regime, 5 *P. citri* larvae were supplied to each Petri dish and food was replaced only every 48 hours. First and second instars of *C. montrouzieri* were placed in groups of 10 in Petri dishes (90 mm × 15 mm); from the third instar on, each predator larva was kept in an individual Petri dish. Upon adult emergence, 30 males and 30 females from each diet regime were collected and their body width and size of genitalia (Fig. 5) were measured using a Zeiss SteREO Discovery V20 stereomicroscope, Zeiss AxioCam HRc camera and AxioVision SE64 software. Body width was measured at the widest width of the elytra. In order to measure the length of the male sipho and female ovipositor, the individuals were killed in ethanol and their abdomen was removed under a Leica S8AP0 stereomicroscope. The abdomen was placed in 10% KOH for 24 hours, after which the genitalia were dissected under a stereomicroscope and rinsed with ethanol. The remaining adults were separated according to sex, and males and females were transferred to separate cages.

As immune activities were considered to be an honest signal for individual attractiveness of male mates, real-time quantitative PCR (RT-qPCR) was used to estimate the expression of immune-related genes, acid phosphatase and pro-phenol oxidase. The two genes can be considered to indicate the intrinsic quality of male mates as they affect their immune function and pathogen resistance^14^,^27^,^28^. Male adults from both types of larval diet regimes were collected from the rearing cages and killed using liquid nitrogen. The extraction of RNA, reverse transcription and RT-qPCR amplification were done according standard procedures^21^,^37^. Briefly, total RNA of adults was extracted using Trizol (Invitrogen), and reverse transcription primed with oligo-dT was used to synthesize cDNA. Three extractions of males from either diet regime were carried out and three replicates of each extraction were used for RT-qPCR. Relative transcript abundance was measured using RT-qPCR on ABI STEPONE PLUS according to the manufacturer’s protocols for SYBR Green. Beta-tubulin (BT) was selected as a reference gene. The primer sequences used in RT-qPCR amplification were as follows: acid phosphatase (Genbank: KR400003): forward 5′ GCCGGAGCGATGATGTC 3′, reverse 5′ TCTGGGAGGCGTCGTAGG 3′; pro-phenol oxidase (Genbank: KR400002): forward 5′ AATAAAGACCGCGAGGCAGAAT 3′, reverse 5′ GGACGCAGTGAGCACCAGTTAG 3′; the reference gene (BT, Genbank: ADI24738.1): forward 5′ CACGGAAGGTACTTGACTGTTG 3′, reverse 5′ GCTGCTGTTCTTGTTTTGGATG 3′.

### Mate choice

In order to examine female mate choice between males from high- or low-nutrition larval diet regimes, we carried out two separate experiments. Adults were provided with ad libitum food (*P. citri* larvae) from emergence to the start of the experiments.

In a first experiment, female mate choice was investigated in the presence of a so-called “monitoring” male. These monitoring males were taken from the groups of adults which had experienced either the high- or low-nutrition larval diet regimes. Each female was placed in a two-compartment Petri dish (90 mm × 15 mm). The compartments were separated by a diametric transparent wall, which allowed the insects to see conspecifics in the other compartment without being able to cross over. Four treatments were carried out. In treatment A, individual females from the high-nutrition diet regime were paired with a male also from the high-nutrition diet regime without another male monitoring from the other compartment. In treatment B, individual females from the high-nutrition diet regime were placed together with a male from the high-nutrition diet regime, but had a monitoring male from the low-nutrition diet regime in the other compartment. In treatment C, individual females from the low-nutrition diet regime were paired with a male also from low-nutrition diet regime without another male monitoring. Finally, in treatment D, individual females from the low-nutrition diet regime were placed together with a male from the low-nutrition diet regime, but had a monitoring male from the high-nutrition diet regime in the other compartment. Each pair was allowed 2 h (14.00–16.00 h) to mate. During this period we recorded mating occurrence, mating latency and mating duration. Thirty replicates were performed for each treatment.

In a second experiment, mate choices of females from the low- and high-nutrition larval diet regime were examined when confronted with two males, one from the low- and one from the high-nutrition larval diet regime. The two males were marked using marker pens (red or white), which have been shown not to influence female mate choice^18^. The two males and the female were then placed together in a non-compartmented Petri dish (15 mm*90 mm) and observed during a 2-h period (14:00–16:00 h). As soon as the female had made a mate choice, the other male was removed. Thereafter, female mating frequency, mating latency and mating duration were recorded. One hundred and twenty replicates were performed for each treatment.

### Reproductive success

Successfully mated females from the second mate choice experiment were singly transferred to individual 90 mm Petri dishes and subjected to either a high- or a low-nutrition adult diet regime. In the high-level adult diet regime, 10 adult *P. citri* (ca. 2.5 mm long) were supplied and refreshed daily; in the low-level diet regime, only 5 adult *P. citri* were supplied and diet was replaced every 48 hours. Eight treatments were done as laid out in (Table 4). For each treatment, we recorded egg production, egg hatchability and egg size within a one-month period. There were 15 replicates for each treatment.

### Analysis

Statistical analysis was performed using SPSS 21 (IBM SPSS Statistical, Chicago, USA). Mating frequency and hatching rates were considered to be binary data, enabling us to calculate standard errors^38^. The phenotypic traits of adults, including body width of males and females and the size of their genitalia were analyzed by one-way analysis of variance (ANOVA). Quantitative expression of acid phosphatase and pro-phenol oxidase was also compared using ANOVA. The females’ sexual selection parameters (mating latency, mating duration and mating frequency) were examined using ANOVA followed by Tukey’s test. All datasets were first tested for normality and homogeneity of variances by a Shapiro-Wilk test and Levene test, respectively. In order to examine the effects of mate selection and food regimes on reproductive performance, we used a General Linear Model (GLM). In this model, reproductive performance parameters (egg production, egg hatch and egg size) were the dependent variables. Diet regimes during adulthood and female body size reflecting the larval nutritional conditions were selected as fixed factors. Male body size, mating latency and mating duration were selected as covariates. We also examined the interaction effects between female size and adult diet regimes on reproductive performance. The significance level of all tests was set at p ≤ 0.05.

## Additional Information

**How to cite this article**: Xie, J. *et al.* Nutrition-dependent phenotypes affect sexual selection in a ladybird. *Sci. Rep.* **5**, 13111; doi: 10.1038/srep13111 (2015).

## Figures and Tables

**Table 1:** Effects of adult food regime, female size, male size, mating latency and copulation duration on egg production as estimated by a General Linear Model (GLM).

	Trait	SS	df	F	P-value
**Egg production**	Nutrition	15103	1	16.681	<0.001^**^
	Female size	25155.727	1	27.785	<0.001^**^
	Male size	312.121	1	0.345	0.558
	Mating latency	957.035	1	1.057	0.305
	Copulation duration	4191.576	1	4.63	0.032^*^
	Female size*Nutrition	59.14	1	0.065	0.799
	Male size*Mating latency*Copulation duration	6111.488	1	6.75	0.01*
	Female size*Nutrition*Male size*Mating latency*Copulation duration	3647.67	3	1.343	0.261
	Error	207333.269	229	905.385	
	Total	1286089	240		

**Table 2:** Effects of adult food regime, female size, male size, mating latency and copulation duration on egg hatch as estimated by a General Linear Model (GLM).

	Trait	SS	df	F	P-value
Egg hatch	Nutrition	0	1	0.024	0.878
	Female size	0	1	0.468	0.495
	Male size	0	1	0.273	0.602
	Mating latency	0.002	1	1.595	0.208
	Copulation duration	0.004	1	3.412	0.066
	Female size*Nutrition	0.002	1	1.899	0.17
	Male size*Mating latency*Copulation duration	0.004	1	3.792	0.053
	Female size*Nutrition*Male size*Mating latency*Copulation duration	0	3	0.127	0.944
	Error	0.239	229	0.001	
	Total	205.601	240		

**Table 3:** Effects of adult food regime, female size, male size, mating latency and copulation duration on egg size as estimated by a General Linear Model (GLM).

	Trait	SS	df	F	P-value
**Egg size**	Nutrition	4.187	1	0.875	0.35
	Female size	2.279	1	0.476	0.491
	Male size	8.701	1	1.819	0.179
	Mating latency	9.315	1	1.948	0.164
	Copulation duration	4.095	1	0.856	0.356
	Female size*Nutrition	5.273	1	1.103	0.295
	Male size*Mating latency*Copulation duration	7.985	1	1.669	0.198
	Female size*Nutrition*Male size*Mating latency*Copulation duration	3.568	3	0.746	0.526
	Error	1095.25	229	4.783	
	Total	1127.496	240		

**Table 4:** Treatments of the experiment measuring reproductive success of *C. montrouzieri* as affected by larval and adult food regimes of females and males.

Adult diet regimes	Female × Male combinations (Larval diet regimes*)			
	F_H_x M_H_	F_H_x M_L_	F_L_x M_H_	F_L_x M_L_
High	HH	HL	LH	LL
Low	HH	HL	LH	LL

*F, female; M, male; L, low-nutrition larval diet regime; H, high-nutrition larval diet regime.
